# Type III secretion system expression in oxygen-limited *Pseudomonas aeruginosa* cultures is stimulated by isocitrate lyase activity

**DOI:** 10.1098/rsob.120131

**Published:** 2013-01

**Authors:** Jade C. S. Chung, Olena Rzhepishevska, Madeleine Ramstedt, Martin Welch

**Affiliations:** 1 Department of Biochemistry, University of Cambridge, Cambridge CB2 1QW, UK; 2 Department of Chemistry, Umeå University, 90187, Umeå, Sweden

**Keywords:** type III secretion, glyoxylate shunt, *Pseudomonas aeruginosa*, anaerobic, isocitrate lyase

## Abstract

*Pseudomonas aeruginosa* is an opportunistic human pathogen and a common cause of chronic infections in individuals with cystic fibrosis (CF). Oxygen limitation was recently reported to regulate the expression of a major virulence determinant in *P. aeruginosa*, the type III secretion system (T3SS). Here, we show that expression of the T3SS in oxygen-limited growth conditions is strongly dependent on the glyoxylate shunt enzyme, isocitrate lyase (ICL; encoded by *aceA*), which was previously shown to be highly expressed in CF isolates. ICL-dependent regulation of the T3SS did not alter the expression level of the master transcriptional regulator, ExsA, but did affect expression of the T3 structural proteins, effectors and regulators (ExsC, ExsD and ExsE). An *aceA* mutant displayed enhanced biofilm formation during anaerobic growth, which suggested that AceA-dependent modulation of type III secretion might impinge upon the RetS/LadS signalling pathways. Indeed, our data suggest that RetS is able to mediate some of its effects through AceA, as expression of *aceA in trans* partially restored T3SS expression in a *retS* mutant. Our findings indicate that AceA is a key player in the metabolic regulation of T3SS expression during oxygen-limited growth of *P. aeruginosa*. To the best of our knowledge, this is the first demonstration that the T3SS can be regulated by factors that do not affect ExsA expression levels.

## 2. Introduction

*Pseudomonas aeruginosa* is an opportunistic human pathogen and a major cause of chronic infections in individuals with cystic fibrosis (CF). *P. aeruginosa* expresses a highly regulated protein secretion machinery called the type III secretion system (T3SS), which translocates a specific subset of exotoxin effector proteins (ExoS or ExoU, ExoT and ExoY) directly into the host cell cytosol where they mediate their pathogenic effects [1–5]. The T3SS regulon consists of approximately 40 coordinately regulated genes which are arranged into five operons, encoding the needle complex, translocation apparatus and regulator proteins; the effectors and chaperones are encoded elsewhere on the genome [6]. Transcription of the regulon is directly coupled to secretion through a cascade of interactions involving ExsC, ExsD and ExsE, which ultimately impinge upon the master transcriptional activator of T3SS gene expression, ExsA [6–10]. When the T3SS is inactive, ExsE and ExsC form a complex, allowing ExsD to bind to and sequester ExsA. When type III secretion (T3S) is induced (e.g. by host cell contact or low Ca^2+^ concentration, established triggers of secretion [6,11]), ExsE is exported through the secretion machinery. This allows ExsC to bind ExsD, liberating ExsA to initiate transcription of the T3SS genes [9,10,12–14].

In recent years, metabolism has been implicated in the regulation of *exsA*-dependent T3SS expression. Rietsch & Mekalanos [15] showed that in metabolically unfavourable conditions, low Ca^2+^-induced *exoS* expression was disrupted in an *aceE* mutant (*aceE* encodes the E1 subunit of pyruvate dehydrogenase (PDH), which controls the flux of glycolytic carbon entering the tricarboxylic acid (TCA) cycle). The opposite effect was observed in mutants in the citrate synthases (*gltA* and *prpC*). Similarly, Dacheux and colleagues [16] showed that PDH mutants in the *P. aeruginosa* clinical CF isolate, CHA, were no longer able to induce *exsA*-dependent T3S or cytotoxicity against polymorphonuclear neutrophils in low Ca^2+^ conditions. (Note that in these early papers describing *aceE* mutants, the PDH-encoding gene was often denoted ‘*aceA*’. However, in the intervening years, the gene annotation has changed and this is not the same as the isocitrate lyase (ICL)-encoding *aceA* gene described in this work, which has not previously been reported to regulate T3S.)

Our previous work suggested that anaerobiosis plays a role in regulating T3SS expression in *P. aeruginosa*, which may be relevant in the oxygen-limited environment of chronic CF lung infections [17]. In support for this, T3SS expression and activity were recently shown to be upregulated when planktonic cultures were grown in low oxygen conditions [18]. Microaerobic (or ‘oxygen-limited’) respiration has been proposed as the dominant growth mode of *P. aeruginosa* in the CF lung and involves the use of three terminal oxidases: two cytochrome *cbb_3_*-type oxidases (Cbb3-1 and Cbb3-2) and a cyanide-insensitive oxidase, CIO [19–21]. Denitrification, involving the use of alternative electron acceptors such as nitrate, has also been demonstrated to occur in these conditions [22]. Given the known link between metabolism and T3S in aerobic growth conditions [15,23], we hypothesized that the re-wiring of central metabolic fluxes during the transition from aerobic to oxygen-limited growth might also impact on T3SS expression in *P. aeruginosa*. In this report, we describe a major new metabolic input regulating oxygen-limited T3SS expression which may play a role in chronic CF lung infections.

## 3. Results

### 3.1. Type III secretion system expression and the glyoxylate shunt pathway in oxygen-limited conditions

**Figure 1.**
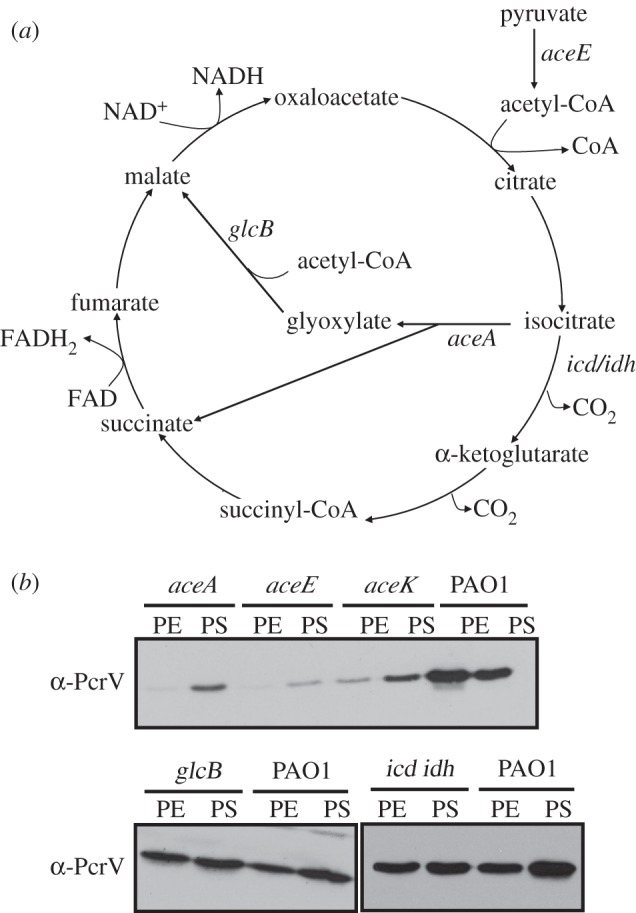
T3SS expression in mutants of the TCA cycle and glyoxylate shunt pathway. (*a*) Diagram depicting the TCA cycle and glyoxylate shunt pathway. (*b*) Western blot analysis of PcrV expression in oxygen-limited planktonic cultures of *P. aeruginosa* PAO1 and the indicated TCA or glyoxylate cycle mutants (sampled at the exponential (PE; 12 h post-inoculation) and stationary (PS; 30 h post-inoculation) phases of growth). Note that as the *aceE* mutant had a growth defect, PE and PS samples were collected at 24 and 48 h post-inoculation, respectively.

One of the most significant central metabolic changes that accompany the onset of low oxygen conditions is the re-routing of carbon skeletons through the glyoxylate shunt pathway. This bypasses the CO_2_-producing steps of the TCA cycle and therefore conserves carbon skeletons for anabolic consumption (figure 1*a*). In the glyoxylate shunt, isocitrate is cleaved by ICL into glyoxylate and succinate. The glyoxylate is condensed with acetyl-CoA to yield malate in a reaction catalysed by malate synthase (MS). This pathway is known to be strongly induced in *P. aeruginosa* during anaerobic growth and during growth on C_2_-sources, such as acetate or fatty acids, as the sole carbon source [24–26]. The alternative catabolic fate of isocitrate is oxidative decarboxylation to yield α-ketoglutarate, a reaction catalysed by isocitrate dehydrogenase. Unlike most Gram-negative bacteria, *P. aeruginosa* contains two divergently transcribed isocitrate dehydrogenase-encoding genes; *icd* (PA2623) and *idh* (PA2624). Both genes are simultaneously expressed in *P. aeruginosa* in AGSY medium (H. G. Stickland & M. Welch 2009, unpublished data). The decision as to whether isocitrate is routed through the glyoxylate shunt or the oxidative decarboxylation steps of the TCA cycle is partly determined by AceK-dependent phosphorylation (and consequent inhibition) of isocitrate dehydrogenase [27].

To investigate the effect of these main metabolic steps on T3SS expression in oxygen-limited conditions, the expression of PcrV (a core component and functional indicator of the T3SS) was analysed in isogenic mutants disrupted in *aceE* (encoding the PDH E1 subunit), *aceA* (encoding ICL), *glcB* (encoding MS), *aceK* (encoding isocitrate dehydrogenase kinase/phosphatase) and an *icd–idh* double mutant (encoding the isocitrate dehydrogenases).

It has previously been shown that T3S is greatly reduced in *aceE* mutants during aerobic growth [15,16]. We found that this was also the case during growth in oxygen-limited conditions (figure 1*b*). Interestingly, a mutant in *aceA* also showed greatly diminished PcrV expression, whereas a mutant in *glcB* (the next enzyme along the glyoxylate shunt pathway) displayed essentially wild-type levels of expression. The *aceK* mutant also had lower PcrV expression (figure 1*b*). In *Escherichia coli*, *aceK* mutants are unable to grow on acetate as a sole carbon source, indicating that phosphorylation of isocitrate dehydrogenase is a prerequisite for redirection of flux through the glyoxylate shunt pathway [27]. Conversely, the *icd–idh* double mutant, in which isocitrate is forced through the glyoxylate shunt, displayed wild-type levels of PcrV expression. To further confirm this observation, we also analysed the transcriptional activity of *pG* (the T3SS operon encoding *pcrGVH-popBD*) in the *icd–idh* double mutant and found that it was comparable with wild-type (see the electronic supplementary material, figure S2*a*). Together, these data indicate that the branch point at which carbon skeletons are routed through either the oxidative decarboxylation steps of the TCA cycle or through the glyoxylate shunt plays a key role in regulating T3S during oxygen-limited growth.

### 3.2. Isocitrate lyase activity is necessary for optimal type III secretion system expression under oxygen-limited conditions

**Figure 2.**
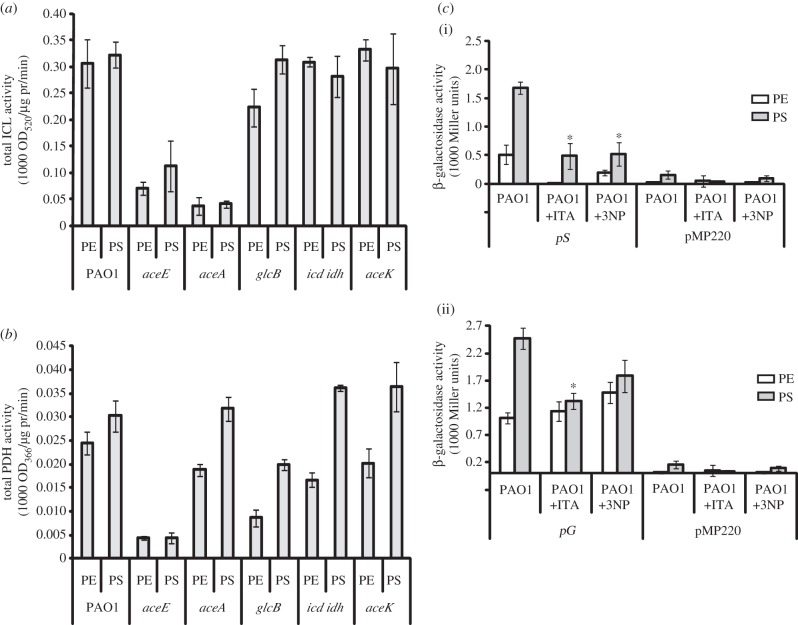
Effect of ICL activity on T3SS expression. Oxygen-limited planktonic cultures of PAO1 and the indicated TCA or glyoxylate cycle mutants (sampled at PE and PS) were analysed for (*a*) total isocitrate lyase (ICL) activity, and (*b*) total pyruvate dehydrogenase (PDH) activity. pr, protein. (*c*) Transcriptional activities of (i) *pS* (pSB307) and (ii) *pG* (pSB308) in PAO1 cultures supplemented with 15 mM itaconic acid (ITA) or 1 mM 3-nitropropionic acid (3NP); pMP220 acted as an empty plasmid control. *p < 0.05.

We next examined the link between T3SS expression and the branchpoint of the TCA and glyoxylate cycles by assaying for *aceA*-encoded ICL and *aceE*-encoded PDH enzyme activities in the oxygen-limited cultures of the wild-type and each mutant (figure 2*a*,*b*, respectively). The *aceE* mutant exhibited low ICL activity, which was comparable with that assayed in the *aceA* mutant (figure 2*a*). Conversely, although PDH activity was negligible in the *aceE* mutant as expected, it was essentially unaffected (relative to the wild-type) in the *aceA* mutant (figure 2*b*). In addition, *glcB*-encoded MS activities in the *aceA* and *aceE* mutants were comparable with that of the wild-type (data not shown). It therefore seems possible that the diminished T3SS expression observed in the *aceE* mutant may in fact be because of its lower ICL activity in these growth conditions. The measured ICL activity in the *aceK* mutant was comparable with that in the wild-type, which was expected since ICL activity is not affected by AceK.

To verify that this effect was associated with the ICL (i.e. enzymatic) activity of the AceA protein, we examined whether the ICL inhibitors itaconic acid and 3-nitropropionic acid could phenotypically mimic the effects of an *aceA* mutation on the transcription of *pS* (encoding *exoS*, a T3 effector) and *pG* (the T3SS operon encoding *pcrGVH-popBD*) in the wild-type background. Indeed, addition of these compounds to oxygen-limited wild-type cultures resulted in a reduction in transcription of both *pS* (figure 2*c*(i)) and *pG* (figure 2*c*(ii)). In conclusion, these data suggest that in oxygen-limited growth conditions, *aceA*-encoded ICL activity is required for optimal T3SS expression.

### 3.3. Type III secretion system expression in an *aceA* mutant under oxygen-limited conditions

**Figure 3.**
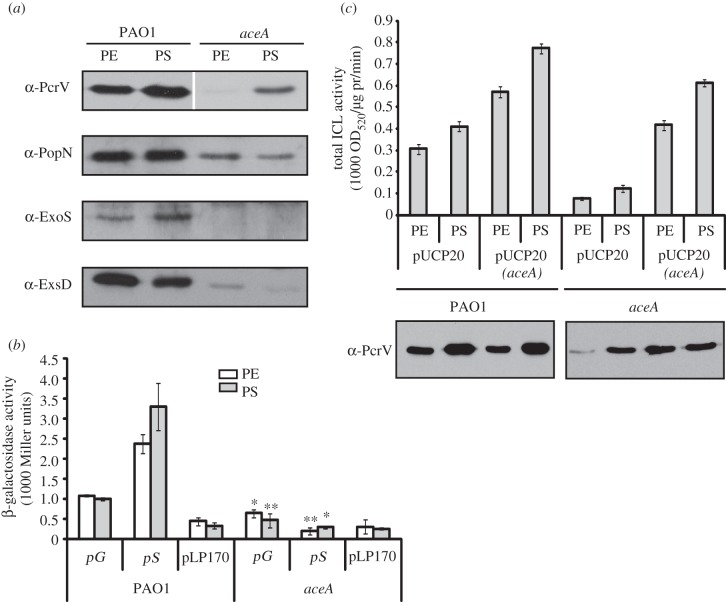
T3SS expression in an *aceA* mutant. T3SS expression in PAO1 and an *aceA* mutant was investigated by analysing oxygen-limited planktonic cultures (sampled at PE and PS) for the following: (*a*) T3SS proteins (PcrV, PopN, ExoS and ExsD), as determined by Western blot analysis; (*b*) transcriptional activities of *pG* (pJC9), *pS* (pJC8) and pLP170 (empty plasmid); (*c*) total ICL activity and corresponding PcrV expression in PAO1 or *aceA* carrying a plasmid-encoded *aceA* gene (pJC10) or the empty plasmid (pUCP20). pr, protein. *p < 0.05; **p < 0.02.

We next analysed the expression of other T3SS-related gene products in an *aceA* mutant. In addition to PcrV, the expression of PopN (a regulator of the T3SS translocation process), ExoS (a T3 effector protein) and ExsD (a T3S regulator) was also greatly reduced in the *aceA* mutant (figure 3*a*). Interestingly, although all of these proteins showed a diminished expression in the *aceA* mutant, some were affected more than others, suggesting that the loss of AceA differentially impacts on these T3SS components. Consistent with these observations, transcription of *pS* (encoding *exoS*) and *pG* (the T3SS operon encoding *pcrGVH-popBD*) was lower in the *aceA* mutant (figure 3*b*). We were also able to show that expression of *aceA* from a plasmid *in trans* could restore PcrV expression and ICL activity in the *aceA* mutant (figure 3*c*).

Given that the enzymatic activity of AceA was important for expression of the T3SS, we also tested whether the products of ICL activity might be accountable. However, when we supplemented oxygen-limited cultures of the *aceA*, *aceE* and *aceK* mutants with succinate or glyoxylate (or indeed with other TCA cycle intermediates, including citrate, isocitrate, α-ketoglutarate, fumarate and glycine (the transamination product of glyoxylate)), PcrV expression was not restored to wild-type levels (see the electronic supplementary material, figure S2*b*) despite (in some cases) improved growth in the presence of the additive.

### 3.4. Effect of an *aceA* mutation on *exsA*, *exsC*, *exsD* and *exsE* expression

**Figure 4.**
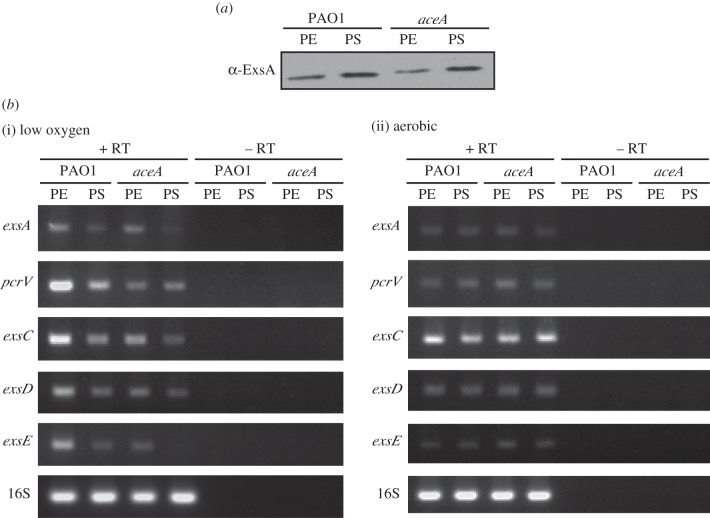
Expression of the master T3 transcriptional regulator (*exsA*) and other T3S regulators (*exsC*, *exsD* and *exsE*) in an *aceA* mutant. The origin of diminished T3SS expression in the *aceA* mutant was investigated: (*a*) ExsA protein expression in PAO1 and an *aceA* mutant in oxygen-limited conditions (sampled at PE and PS), as determined by Western blot analysis; (*b*) RT–PCR analysis of T3SS gene transcripts in PAO1 and *aceA*: total RNA was isolated from cultures grown under (i) oxygen-limited or (ii) aerobic conditions at PE and PS. 16S rRNA (16S) transcript levels indicate equal loading. Samples lacking RT show that no DNA contamination was present.

Since the activity of more than one unlinked T3SS gene was depressed in an *aceA* mutant (figure 3*a*), it seemed probable that the master transcriptional regulator of T3SS expression, ExsA, might be involved. However, ExsA protein levels in the *aceA* mutant were found to be similar to wild-type (figure 4*a*). Furthermore, when *exsA* transcript levels were measured by reverse transcriptase (RT)–PCR in the oxygen-limited wild-type and *aceA* mutant cultures, they were found to be comparable (figure 4*b*(i)). Conversely, and in agreement with protein expression and transcriptional activity (figure 3*ab*, respectively), *pcrV* transcript levels were much lower in the *aceA* mutant at the exponential (PE) and stationary (PS) growth phases relative to wild-type (figure 4*b*(i)). Note that T3 transcript levels were higher during PE oxygen-limited growth compared with PS, whereas the opposite was observed for protein expression. We speculate that this may be because of the (as yet uncharacterized) post-transcriptional regulatory processes that impact upon the expression levels of T3SS proteins in the different growth phases.

The levels of ‘free’ ExsA in the cell are tightly controlled by a series of regulatory proteins, namely ExsC, ExsD and ExsE. RT–PCR revealed that the transcript levels for all three regulators were reduced in the *aceA* mutant at both growth phases relative to wild-type (figure 4*b*(i)). Notably, although ExsA levels remained unchanged in the *aceA* mutant, levels of *exsD* (which encodes an anti-activator of ExsA) mRNA (figure 4*b*(i)) and protein (figure 3*a*) were much lower in the mutant. Based on the current model in which expression of the T3SS is solely controlled by the level of free ExsA, a reduction in ExsD expression should give rise to enhanced expression of the T3SS proteins [9,10,12–14]. However, the opposite was observed. Clearly, the existing model is an over-simplification that does not account for the regulation of T3SS expression by metabolic inputs during oxygen-limited growth.

Consistent with the notion that oxygen limitation stimulates T3S, *pcrV* transcript levels were significantly higher in wild-type cultures grown under oxygen-limited conditions (figure 4*b*(i)) than under aerobic conditions (figure 4*b*(ii)) at both growth phases. Furthermore, no differences in T3 transcript levels were observed between the wild-type and *aceA* mutant under aerobic conditions (figure 4*b*(ii)), despite comparable growth (see the electronic supplementary material, figure S1*b*), suggesting that regulation by *aceA* is specific to oxygen-limited conditions.

### 3.5. Biofilm formation is enhanced in an *aceA* mutant and can be complemented by expression of *aceA in trans*

**Figure 5.**
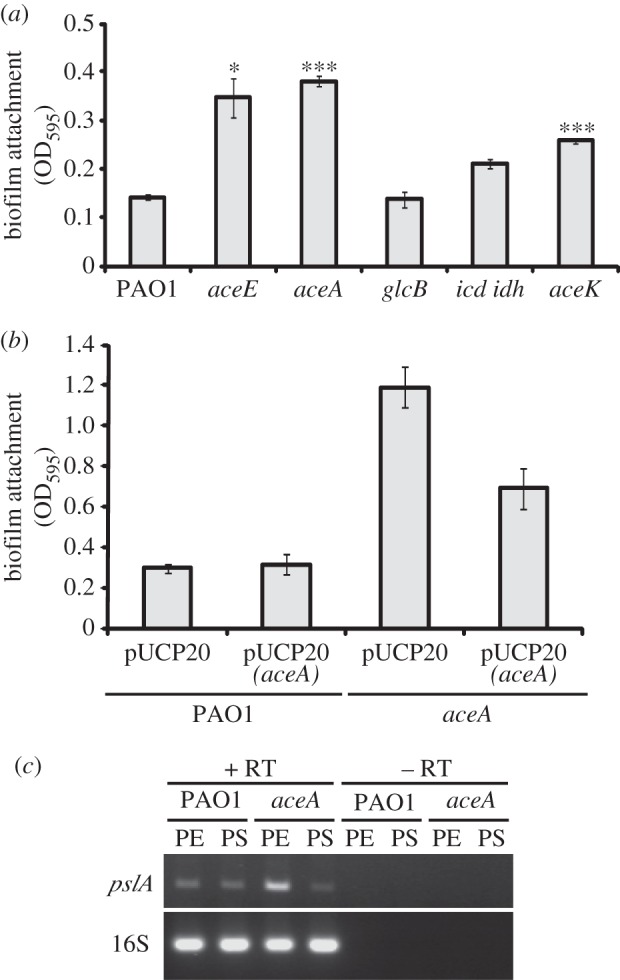
Biofilm formation in TCA and glyoxylate cycle mutants under anaerobic conditions. Anaerobic biofilm formation in (*a*) PAO1 and the indicated TCA and glyoxylate cycle mutants, and (*b*) PAO1 and an *aceA* mutant carrying a plasmid-encoded *aceA* gene (pJC10) or the empty plasmid (pUCP20). *p < 0.05; ***p < 0.01. (*c*) Expression of the *pelA* gene transcript in PAO1 and an *aceA* mutant, as analysed by RT–PCR analysis of total RNA isolated from oxygen-limited cultures at PE and PS. 16S rRNA (16S) transcript levels indicate equal loading. Samples lacking RT show that no DNA contamination was present.

The CF lung has been demonstrated to be an oxygen-limited environment, where *P. aeruginosa* is thought to grow as biofilm-like aggregates [28]. Given our earlier findings suggesting that anaerobiosis-mediated T3SS expression occurred in biofilms [17], we next examined whether biofilm formation might be affected in the *aceA*, *aceE*, *aceK*, *icd–idh* and *glcB* mutants. Biofilm formation under anaerobic conditions was significantly enhanced in the *aceA* mutant as well as in the other mutants that showed reduced PcrV expression (figure 5*a*). Furthermore, the enhanced biofilm formation by the *aceA* mutant could be restored towards the wild-type situation by expression of *aceA in trans* from a plasmid (figure 5*b*). RT–PCR analysis of a transcript (*pslA*) encoding an enzyme involved in the biosynthesis of an extracellular polysaccharide associated with biofilm matrix formation, Psl, showed that the levels of *pslA* were increased in an *aceA* mutant at the exponential growth phase relative to wild-type (figure 5*c*). These results suggest that *aceA* can regulate biofilm formation by influencing *psl* expression.

### 3.6. Expression of *aceA* partially bypasses the type III secretion defect in a *retS* mutant

The inverse relationship between biofilm formation and T3S in the *aceA* mutant was reminiscent of the phenotype associated with a *retS* mutation. Furthermore, Hagins *et al*. [29] previously reported that *aceA* transcription is repressed in a *retS* mutant. We therefore investigated whether AceA might contribute towards the *retS* phenotype.

As a first step, we confirmed that the RetS signalling pathway was also necessary for the reciprocal regulation of T3SS expression and biofilm formation in oxygen-limited conditions (see the electronic supplementary material, figure S3a,b). This was indeed the case. Note that in contrast to the situation in the *aceA* mutant, *exsA* transcript levels (as well as the transcript levels for *exsC*, *exsD* and *exsE*) were lower in a *retS* mutant relative to wild-type (see the electronic supplementary material, figure S3b; cf. figure 4*b*(i)).

**Figure 6.**
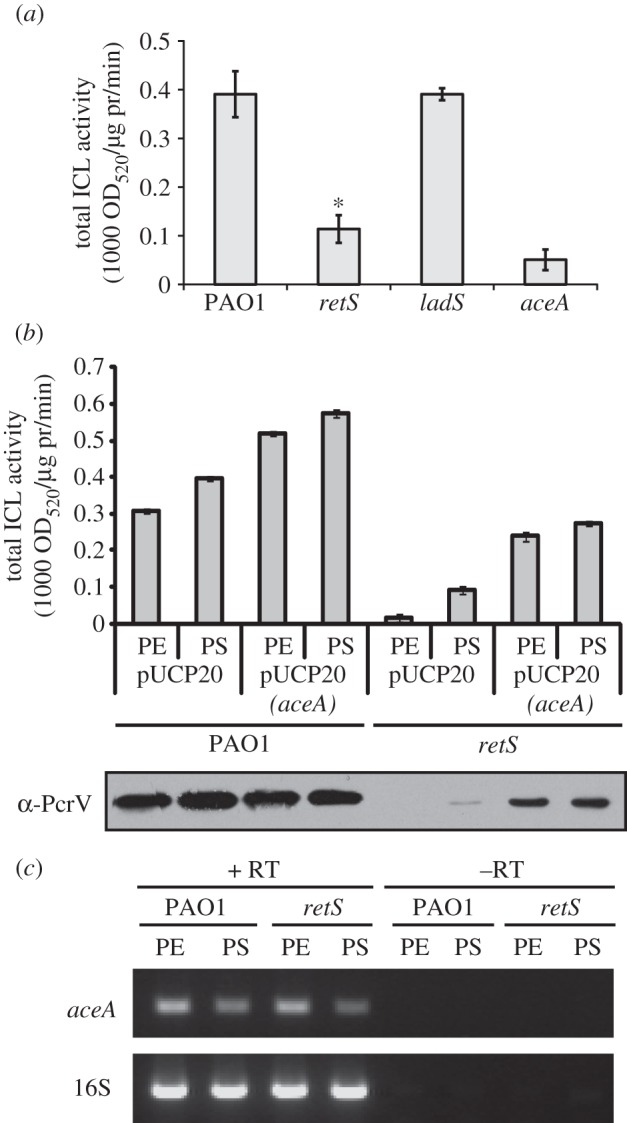
Effect of ICL activity on the RetS signalling pathway. T3SS expression and ICL activity were investigated in a *retS* mutant grown under oxygen-limited conditions. (*a*) Total ICL activity in PAO1 and mutants in *retS*, *ladS*, and *aceA* (harvested at PS). *p < 0.05. (*b*) Total ICL activity and corresponding PcrV expression in PAO1 or in a *retS* mutant carrying a plasmid-encoded *aceA* gene (pJC10) or empty plasmid control (pUCP20), as indicated, at PE and PS. (*c*) RT–PCR analysis of the *aceA* gene transcript in PAO1 and a mutant in *retS*. Total RNA was isolated from cultures grown under oxygen-limited conditions at PE and PS. 16S rRNA (16S) transcript levels indicate equal loading. Samples lacking RT show that no DNA contamination was present.

ICL activity was found to be depressed in a *retS* mutant (figure 6*a*). Furthermore, expression of *aceA* from a plasmid *in trans* led to partial complementation of ICL activity and PcrV expression in the *retS* mutant (figure 6*b*). These observations indicate that the T3S defect associated with a *retS* mutant could be at least in part attributed to the lower *aceA* activity. To determine if *retS* may affect T3SS by modulating *aceA* expression, RT–PCR analysis was carried out. However, and in contrast to the results of Hagins *et al*. [29], who studied *aceA* expression levels in aerobically grown cultures, *aceA* transcript levels in oxygen-limited cultures of the *retS* mutant were found to be similar to those in the wild-type (figure 6*c*). Therefore, the depressed ICL activity associated with the *retS* mutant in oxygen-limited conditions is because of post-transcriptional effects.

T3SS expression and biofilm formation are reciprocally regulated by the RetS/LadS signalling pathways through a complex mechanism involving the GacS/GacA two-component system, the small regulator RNAs RsmZ and RsmY, and the translational repressor RsmA [30–34]. Activation of LadS (or reduced expression of RetS) results in the formation of GacS homodimers, which leads to the phosphorylation and activation of GacA and concomitant production of *rsmZ* and *rsmY*. These small RNAs bind to and sequester RsmA, which in turn leads to the activation of biofilm formation [32,33]. Conversely, when RetS is activated, it forms heterodimers with GacS, thus preventing stimulation of the GacA/GacS signalling pathways. Free RsmA binds to and influences the stability of specific mRNA targets, which indirectly leads to the activation of *exsA*-dependent T3SS expression [33].

**Figure 7.**
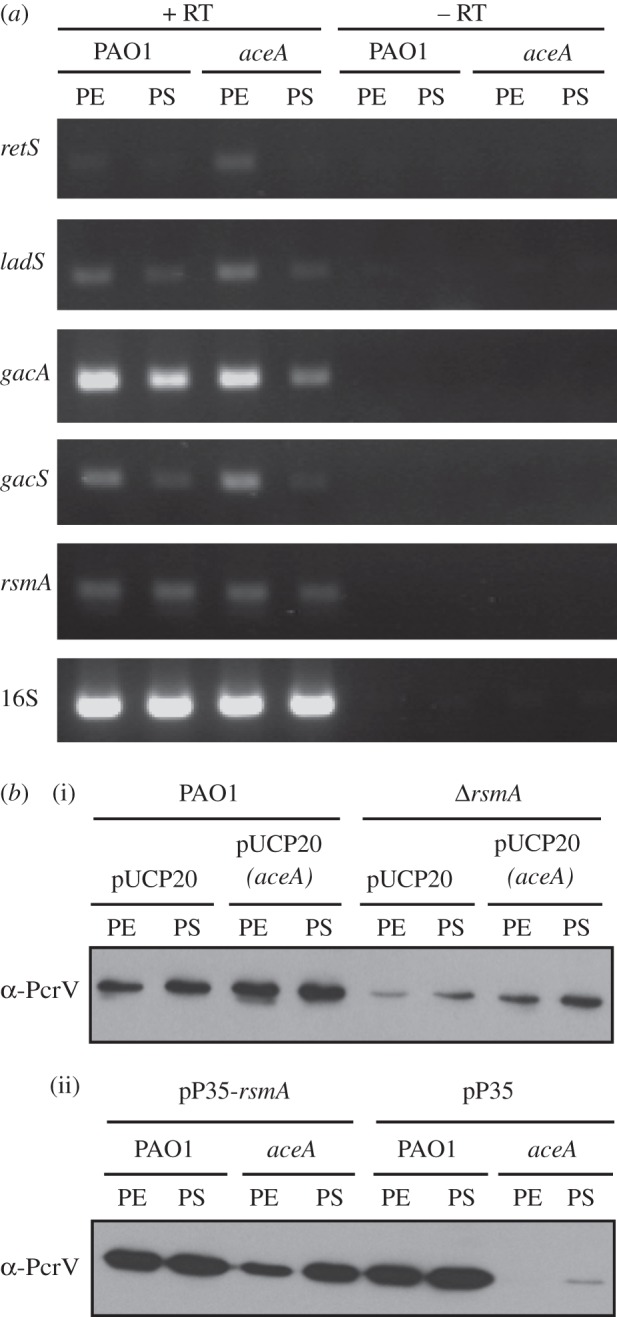
Expression of the RetS signalling pathway in an *aceA* mutant. (*a*) RT–PCR analysis of the indicated gene transcripts in PAO1 and an *aceA* mutant. Total RNA was isolated from cultures grown under oxygen-limited conditions at PE and PS. 16S rRNA (16S) transcript levels indicate equal loading. Samples lacking RT show that no DNA contamination was present. (*b*) PcrV expression in (i) PAO1 and a *ΔrsmA* mutant carrying a plasmid-borne *aceA* gene (pJC10) or empty plasmid control (pUCP20), and (ii) PAO1 and an *aceA* mutant carrying a plasmid-borne *rsmA* gene (pP35-*rsmA*) or empty plasmid control (pP35), as indicated.

RT–PCR was used to assess whether the expression level of *retS*, or other components of the signalling pathway were affected in the *aceA* mutant (figure 7). Transcript levels for *gacA*, *gacS* and *rsmA* were all similar between the wild-type and the *aceA* mutant. Transcript levels for *retS* and *ladS* appeared to be higher in the *aceA* mutant during the exponential phase (PE) of growth. This may indicate that some sort of feedback mechanism is in action, perhaps compensating for the lower T3SS expression in the *aceA* mutant. However, it is also possible that the increased expression of *retS* in this mutant may be cancelled out by the concomitantly increased expression of *ladS*.

Consistent with the central role played by RsmA in regulating T3S, an *rsmA* mutant displayed reduced PcrV expression relative to the wild-type (see the electronic supplementary material, figure S3*a*(ii)). However, and unlike the *retS* mutant, when *aceA* was expressed *in trans* in the *rsmA* mutant, only a marginal increase in PcrV expression was observed (figure 7*b*(i)). In contrast, the low level of PcrV expression in the *aceA* mutant could be complemented by expressing *rsmA* from a plasmid *in trans* (figure 7*b*(ii)). Taken together, these data indicate that AceA primarily exerts its effect(s) on T3S downstream of RetS but upstream of RsmA.

## 4. Discussion

Recent studies indicate that T3SS expression in *P. aeruginosa* is activated upon exposure to low oxygen conditions [17,18]. In the present report, we show that an enzyme involved in anaerobic metabolism, ICL (encoded by *aceA*), plays an important role in ensuring optimal T3SS expression under these growth conditions. Loss of *aceA* function had no effect during aerobic growth. An *aceA* mutant also displayed enhanced biofilm formation during anaerobic growth, which suggested that AceA might modulate these activities through the RetS/LadS signalling pathways. Indeed, expression of *aceA in trans* was able to partially complement T3SS expression in a *retS* mutant. In contrast, expression of *aceA in trans* did not substantially restore PcrV expression in an *rsmA* mutant. This suggests that AceA primarily impinges upon T3S upstream of RsmA, and is necessary for maximal T3SS expression when the RetS signalling pathway is activated in oxygen-limited conditions.

Our results provide further insight into the link between central carbon metabolism and T3SS expression, as previously reported by Rietsch & Mekalanos [15]. However, the mainstream model for T3SS regulation posits that control is primarily achieved through modulation of *exsA* expression (ExsA is the master T3 transcriptional regulator). In contradiction with this model, we found that protein and transcript levels of ExsA were unaffected in the *aceA* mutant, in spite of the severely reduced level of T3SS protein expression. Moreover, the expression of ExsD (an anti-activator of T3SS expression) was *decreased* in the *aceA* mutant. ExsD is thought to act by binding/sequestering ExsA, so this lower expression of ExsD should *increase* the level of ‘free’ ExsA and concomitantly increase T3SS expression. Since the opposite was observed, it seems that under oxygen-limiting growth conditions, metabolic regulation of the T3SS involves more than simple modulation of free ExsA levels.

*aceA* (PA2634) is a monocistronic gene of the glyoxylate shunt pathway and is the only ICL-encoding gene in *P. aeruginosa* PAO1 [35,36]. ICL expression is stimulated during growth in oxygen-limiting conditions, and, consistent with the anaerobic nature of the CF lung, the enzyme was found to be highly expressed in *P. aeruginosa* isolates from chronically infected CF individuals [24,25,29,37]. Although its primary function in *P. aeruginosa* is carbon catabolism, recent evidence suggests that ICL can also have an impact on pathogenesis [36–38]. For example, in the mucoid *P. aeruginosa* CF isolate, FRD1, increased ICL activity was directly linked to the production of a precursor metabolite (glycine) of hydrogen cyanide (HCN), a poison and potent inhibitor of cytochrome *c* oxidase and other metalloenzymes, including nitrite reductase and superoxide dismutase [38,39]. However, as far as we are aware, no previous workers have reported that ICL impinges upon T3SS expression in any organism. Moreover, and unlike many other known modulators of T3SS expression, the regulatory effect of AceA was not routed through the control of *exsA* expression levels. Concomitant with the diminished T3SS expression in the *aceA* mutant, we also observed that this strain exhibited enhanced biofilm formation in oxygen-limited conditions. We previously reported that the T3SS is expressed in PAO1 biofilms, and that this expression correlated with the expression of genes involved in anaerobic respiration [17,40]. We therefore speculate that ICL may play a role in regulating T3SS expression during the development of oxygen-limited conditions inside biofilms.

Our data indicate that regulation by *aceA* may be mediated through the RetS/LadS signalling pathways. O'Callaghan *et al*. [18] recently presented data showing that the T3SS was activated during oxygen-limited growth in LB medium, and suggested a model implicating components of the same signalling pathways. These authors proposed that oxygen limitation is sensed by Anr (anaerobic regulator), which stimulates the expression of *narL* of the NarL/NarX two-component system regulating denitrification and arginine fermentation. In turn, NarL represses expression of the RsmA-antagonistic RNAs, *rsmZ* and *rsmY*. The lower levels of *rsmZY* lead to a higher concentration of free RsmA, which indirectly leads to the stimulation of *exsA*-dependent T3SS expression. However, we found that AceA-dependent modulation of T3S did not involve changes in ExsA transcript or protein levels. Furthermore, in oxygen-limited AGSY media, PcrV expression in a *narL* mutant was comparable with that in the wild-type (J. C. S. Chung & M. Welch, unpublished data). Lastly, in oxygen-limited conditions, the T3S defect associated with a *retS* mutant could be at least partially attributed to the lower ICL activity in this genetic background, since the *retS* T3S phenotype could be bypassed (in proportion with the degree of restoration of ICL activity) by expression of *aceA in trans*. Interestingly, in spite of the expression of *aceA* from a multicopy plasmid, we were never able to completely restore PcrV expression or ICL activity to wild-type levels in the *retS* mutant. In addition, and considering that *aceA* transcript levels were unaffected (relative to the wild-type) in the *retS* mutant, these data indicate either that ICL activity is silenced in the absence of RetS signalling, or that translation of the *aceA* transcript is suppressed.

**Figure 8.**
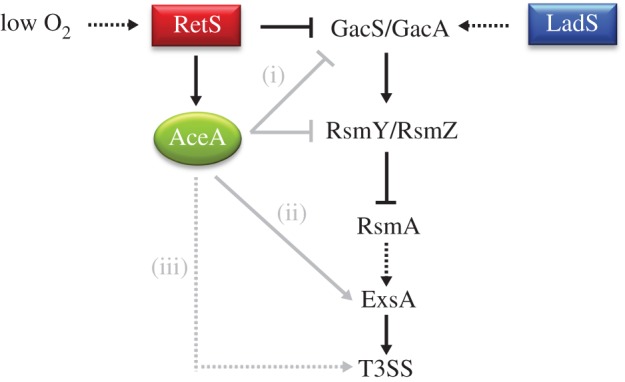
Possible mechanisms for AceA-dependent regulation of T3SS expression in oxygen-limited conditions. AceA-dependent regulation of T3SS expression may involve (i) a direct interaction between AceA and components of the RetS/LadS signalling pathways (e.g. GacS/GacA or RsmY/RsmZ); (ii) modulation of ExsA activity by AceA itself or an AceA-derived metabolite; and/or (iii) direct or indirect modulation of the abundance of one or more metabolites that impinge upon T3SS expression.

At this stage, we can only speculate on the mechanism(s) by which AceA might impact upon T3SS expression. Some possibilities are outlined in figure 8. Our data indicate that during anaerobic growth conditions, AceA impinges on the signalling pathway at some point between RetS and RsmA, so these proteins (and/or GacA/GacS or the small RNAs, *rsmZY*) could directly interact with AceA. However, and mitigating against a direct protein–protein or protein–RNA interaction is the observation that it is ICL *activity* (rather than the presence of the AceA protein *per se*) that is necessary for optimal T3SS expression. Another possibility is that ICL activity may directly or indirectly alter the abundance of one or more metabolites which affect T3SS expression (e.g. through allosteric interactions). If so, the identity of these low molecular weight effectors remains unknown. Alternatively, mutation of *aceA* could lead to large-scale metabolic perturbations that indirectly impact on the T3SS. We feel that this is unlikely because the *aceA* mutant did not display a growth defect (compared with the wild-type) as might be expected if its fitness was impaired by global metabolic ‘rewiring’. Consistent with this, gas chromatography–mass sprectometry profiling of the *aceA* mutant and the wild-type failed to reveal any significant differences in the resolved metabolites (data not shown). Finally, and although ExsA expression was unaffected in the *aceA* mutant, it remains formally possible that AceA (or an AceA-dependent metabolite) may modulate ExsA *activity* (i.e. its ability to bind T3SS promoters or regulatory proteins) through post-translational effects. Post-translational modification of ExsA may be a means to temporally modulate T3S activity such that it becomes integrally linked to the metabolic state of the cell. In this regard, we note that enzyme-catalysed lysine acetylation has recently been shown to regulate the activity of many bacterial proteins, including ICL [41]. Moreover, an activated acetate metabolite (acetyl phosphate) has been implicated in regulation of the T3SS-encoding SPI-1 pathogenicity island in *Salmonella* [42].

In summary, we have demonstrated that the re-routing of metabolic flux through the glyxoylate shunt plays a key role in regulating T3SS expression in oxygen-limited growth conditions. Notably, ICL-dependent metabolic control was found to key into the RetS signalling pathway. This T3S induction by ICL probably contributes to the T3S observed in *P. aeruginosa* biofilms [17,40] and, furthermore, may play an important role during chronic *P. aeruginosa* infections in the CF lung. Future work will focus on understanding the detailed mechanism by which AceA affects T3SS expression during oxygen limitation.

## 5. Material and methods

### 5.1. Bacterial strains, plasmids and growth conditions

Bacterial strains and plasmids used in this study are listed in the electronic supplementary material, table S1. *Pseudomonas aeruginosa* strains were grown in AGSY medium (56 mM alanine, 17 mM K_2_HPO_4_, 86 mM NaCl, 100 μM CaCl_2_, 10 mM MgSO_4_, 5 µM FeCl_2_, 7.5 µM ZnCl_2_, 0.5% (v/v) glycerol, 3 g l^−1^ yeast extract (which contains approx. 20 µM KNO_3_), pH 7) at 37°C. Note that supplementation of oxygen-limited cultures with KNO_3_ (20 mM) resulted in improved growth rates (see the electronic supplementary material, figure S4*a*) but led to no major differences in T3SS protein expression (see the electronic supplementary material, figure S4*b*). Aerobic planktonic cultures were grown with vigorous shaking for 3 h (mid-PE) or 9 h (PS). Oxygen-limited planktonic cultures were grown with a thick (approx. 1 cm) mineral oil overlay and gentle shaking for 12 h (PE) or 30 h (PS). Note that as the *aceE* mutant had a growth defect, PE and PS samples were collected at 24 and 48 h of growth, respectively. *Escherichia coli* strains were grown in Luria broth (LB) at 37°C. Antibiotics were used at final concentrations of 60 µg ml^−1^ tetracycline (Tc), 250 µg ml^−1^ carbenicillin (Cb) or 50 µg ml^−1^ gentamicin (Gm) for *P. aeruginosa*, and 15 µg ml^−1^ Tc, 50 µg ml^−1^ Cb, 10 µg ml^−1^ Gm or 50 µg ml^−1^ kanamycin (Km) for *E. coli*.

### 5.2. Strain and plasmid construction

The *Δicd*–*Δidh* mutant was generated by marker exchange in PAO1. The marker exchange construct was produced by overlap PCR, fusing the 500 bp region upstream of *icd* (icd_500_) with the 500 bp region downstream of *idh* (idh_500_). The resulting construct was digested with BamHI and EcoRI and cloned into pUCP20 to generate pJC3. NdeI–SpeI restriction sites were incorporated between the two fragments to allow the insertion of a gentamicin resistance cassette (a 1 kb fragment obtained by PCR amplification from pTnMod-OGm [43]) yielding pJC4. A 2 kb fragment containing the disrupted *icd–idh* region was excised from pJC4 by digestion with BamHI and EcoRI and ligated to the suicide vector pEX18Tc to yield pJC5. pJC5 was then introduced into PAO1 by a conjugal transfer using CC118λpir as a donor and HH26 (pNJ5000) as a helper strain. Resulting *trans*-conjugants were selected on Gm and cured of the plasmid by counterselection on LB agar containing 10% (w/v) sucrose. Confirmation of the deletion mutant was carried out by PCR and sequencing across the junction regions.

The *ΔgacA* and *ΔrsmA* mutants were generated by marker exchange in PAO1 using the pEXG2-*ΔgacA* [44] and pEXG2-*ΔrsmA* constructs, respectively (kindly provided by Dr Arne Rietsch (Case Western Reserve University, USA)). The marker exchange constructs were introduced into PAO1 by the conjugal transfer using *E. coli* β2163 as a donor. The resulting *trans*-conjugants were selected on Gm and cured of the plasmid by counterselection on LB agar containing 10% (w/v) sucrose. Confirmation of the deletion mutants was carried out by PCR and sequencing.

pJC8 and pJC9 were constructed by insertion of the promoter regions of the *exoS* and *pcrGVH-popBD* operons, respectively, at the SmaI and BamHI restriction sites upstream of the *lacZ* ORF in pLP170. pJC10 was constructed by introducing the PCR-amplified *aceA* ORF and its native promoter into the EcoRI and HindIII restriction sites of pUCP20. pJC8, pJC9 and pJC10 were introduced into PAO1 by electroporation, using the protocol described by Choi *et al*. [45]. Plasmid insertions were verified by PCR and sequencing.

### 5.3. Western blot analysis

Whole-cell lysates from planktonic cultures were prepared by normalizing cell pellets to the same OD_600_ using TE lysis buffer (50 mM Tris, 4 mM EDTA, pH 8.3) and vortexing vigorously. This method was found to yield equivalent protein concentrations across different samples. Whole-cell lysates (20 µl) were separated on 12 per cent sodium dodecyl sulfate–polyacrylamide gels, transferred to polyvinylidene fluoride membrane and analysed by Western blotting. Enhanced chemiluminescence peroxidase labelled anti-rabbit or anti-guinea pig antibodies (Sigma) were used as secondary antibodies. Blots were developed using Immobilon Western Chemiluminescent HRP Substrate (Millipore). All Western analyses were repeated on independent cultures at least three times. Representative results are shown.

### 5.4. β-Galactosidase assay

β-Galactosidase activity was measured essentially as described by Miller [46]. Briefly, cells were permeabilized by treatment with toluene in Z-buffer (60 mM Na_2_HPO_4_, 40 mM NaH_2_PO_4_, 10 mM KCl, 1 mM MgSO_4_.7H_2_O, 50 mM β-mercaptoethanol). β-Galactosidase activity was measured by incubating diluted toluenized cell suspension with 4 mg ml^–1^ *O*-nitrophenyl-β-galactoside (ONPG) at 37°C and monitoring the change in absorbance at 420 nm. Miller units are defined as *Δ*OD_420_ min^−1^ ml^−1^ OD_600_^–1^. All β-galactosidase assays were repeated on independent cultures at least thrice.

### 5.5. Isocitrate lyase activity

ICL activity was assayed based on the method of Diehl & McFadden [47]. Total intracellular proteins were prepared by resuspending cell pellets in TE lysis buffer (50 mM Tris–HCl, 4 mM EDTA, pH 8.3) followed by sonication of the cell suspension and centrifugation at 10 000*g* for 15 min at 4°C to remove cell debris. ICL activity was determined by incubating 50 µg protein extract in ICL buffer (50 mM MOPS, 5 mM MgCl_2_, 1 mM DTT, 0.1% (v/v) phenylhydrazine, 1 mM isocitrate, pH 7.3) for 10 min at 37°C. The reaction was stopped by adding 0.45 ml of concentrated HCl. Potassium ferricyanide (0.25% w/v) was then added to the mixture and incubated at room temperature for 10 min. Glyoxylate production was measured as a colorimetric change at 520 nm, which occurs because of the reaction of ferricyanide with the phenylhydrazone of glyoxylate.

### 5.6. Pyruvate dehydrogenase activity

PDH activity was assayed according to Bourguignon *et al*. [48]. Briefly, 100 µg protein extract was incubated at 30°C in PDH buffer (5 mM MgCl_2_, 0.2 mM CoASH, 0.5 mM thiamine pyrophosphate, 2 mM 3-acetylpyridine adenine dinucleotide (APAD^+^), 2 mM DTT, 15 mM sodium pyruvate, 30 mM potassium phosphate, pH 7) and the formation of APADH (the reduced form) was measured by monitoring the absorbance at 366 nm.

### 5.7. Biofilm attachment assay

For biofilm attachment in aerobic conditions, cells seeded in static 96-well polystyrene microtitre plates were incubated at 37°C for 24 h. For biofilm attachment in anaerobic conditions, plates were incubated in an Anaerogen jar (Oxoid) at 37°C for 3 days. Following incubation, culture supernatant and non-adherent cells were removed and the wells were washed once with 300 µl water. Attached cells were stained for 30 min with 0.1% (w/v) crystal violet. The stained plates were rinsed thrice with water and the adsorbed dye was released by adding 50% (v/v) ethanol. Attachment was quantified by measuring absorbance at 595 nm.

### 5.8. Total RNA isolation and reverse transcriptase–PCR

Cells were collected in RNAlater solution (Ambion) and incubated on ice for 1 h before sedimentation by centrifugation at 3200*g* for 15 min, 4°C. Cell pellets were incubated in 1 mg ml^–1^ lysozyme for 15 min at room temperature and total RNA was extracted using the RNeasy Mini kit (Qiagen) according to the manufacturer's instructions. The resulting RNA (200 ng) was used as a template for reverse transcription and conversion into cDNA. PCR reactions were prepared with 5 µl of diluted cDNA (1 : 5 in water) as a template and primers specific to the specified genes. All RT–PCR analyses were carried out on samples from at least three independent cultures. Representative results are shown.

### 5.9. Statistical analysis

Statistical analyses were performed using the Student's *t*-test based on three independent replicates. A *p*-value <0.05 was considered as statistically significant.

## 6. Acknowledgements

This work was supported by the Biotechnology and Biological Sciences Research Council and by the Isaac Newton Trust (Cambridge). Jade Chung is sponsored by the Leathersellers’ Company in association with Fitzwilliam College (University of Cambridge, UK). Arne Rietsch (Case Western Reserve University, USA) is thanked for provision of the PcrV, ExoS, PopN, ExoT and ExoY antibodies, the pEXG2 series plasmids for constructing the *gacA* and *rsmA* deletion mutants, the pPSV35 over-expression constructs, and for his helpful comments on the manuscript. Sylvie Elsen (INSERM, France) is thanked for the provision of the ExsA and ExsD antibodies. Alain Filloux (Imperial College, London, UK) is thanked for providing the pSB series plasmids for assaying the transcriptional activity of T3 operons. Julian Griffin is thanked for his assistance with metabolite profiling. The authors have no conflicting interests.

## Supplementary Material

Table S1

## Supplementary Material

Figure S1: Growth of TCA and glyoxylate cycle mutants.

## Supplementary Material

Figure S2: T3SS expression and the TCA and glyoxylate cycles.

## Supplementary Material

Figure S3: T3SS expression and the RetS/LadS signalling pathways.

## Supplementary Material

Figure S4: Oxygen-limited cultures supplemented with KNO3.
