# Supplementary material for: Type III secretion system expression in oxygen-limited Pseudomonas aeruginosa cultures is stimulated by isocitrate lyase activity
Source: Open Biol. 2013 Jan;3(1):120131. doi: 10.1098/rsob.120131 (PMC3603453; doi:10.1098/rsob.120131)
Supplement: Table S1 [file rsob120131-s1.doc]

**Table S1**

| **Strain/Plasmid** | **Description** | **Source** |
| --- | --- | --- |
| **Strain**  *P. aeruginosa*  PAO1  *aceA*::Tn*  *aceE*::Tn*  *glcB*::Tn*  *aceK*::Tn*  *retS*::Tn*  *ladS*::Tn*  PAO1 Δ*icd* Δ*idh*  PAO1 Δ*gacA*  PAO1 Δ*rsmA*  *E. coli*  β2163  CC118λpir  HH26 (pNJ5000)  **Plasmids**  pSB307  pSB308  pMP220  pJC3  pJC4  pJC5  pTnMod-OGm  pJC8  pJC9  pLP170  pEX18Tc  pJC10  pUCP20  pPSV35  pP35-*rsmA*  pEXG2  pEXG2-Δ*gacA*  pEXG2-Δ*rsmA* | Wild type (WT)  *aceA*::mini-Tn5-lux mutant of PAO1, Tc^R^  *aceE*::mini-Tn5-lux mutant of PAO1, Tc^R^  *glcB*::mini-Tn5-lux mutant of PAO1, Tc^R^  *aceK*::mini-Tn5-lux mutant of PAO1, Tc^R^  *retS*::mini-Tn5-lux mutant of PAO1, Tc^R^  *ladS*::mini-Tn5-lux mutant of PAO1, Tc^R^  Marker exchange mutant of *icd* and *idh*, Gm^R^  Deletion mutant in *gacA*  Deletion mutant in *rsmA*  (F^−^) RP4-2-Tc::Mu *ΔdapA*::(*erm-pir*), Km^R^ Em^R^  ∆(*ara-leu*), *araD ∆lacX74*, *galK*, *phoA20*, *thi-1*, *rpsE*, *rpoB*, *argE(Am)*, *recA1*, lysogenized with λ*pir* phage  Conjugal transfer parent strain  *pS-lacZ* reporter, Tc^R^  *pG-lacZ* reporter, Tc^R^  *lacZ* transcriptional fusion, Tc^R^  pUCP20 containing the PCR-amplified DNA fragment, -icd_500_-idh_500_-, with internal NdeI-SpeI restriction sites  pJC3 with Gm cassette introduced at the restriction sites  pEX18Tc containing the digested DNA fragment, -icd_500_-Gm-idh_500_-, from pJC4  Tn*Mod* plasposon, Gm^R^  *pS-lacZ* reporter, Cb^R^  *pG-lacZ* reporter, Cb^R^  *lacZ* transcriptional fusion, Cb^R^  Suicide vector for marker exchange, Tc^R^  pUCP20-derived plasmid expressing *aceA* under its native promoter, Cb^R^  Pseudomonas shuttle vector, Cb^R^  Shuttle vector, PA origin, *lacI^q^*, *lacUV5* promoter, MCS of pUCP18, pBR322, Gm^R^  *rsmA* in pPSV35  Allelic exchange vector with pBR origin, *sacB*, Gm^R^  pEXG2 with flanking regions to introduce an unmarked *gacA* deletion  pEXG2 with flanking regions to introduce an unmarked *rsmA* deletion | B. Iglewski (Uni. of Rochester, USA)  [49]  [49]  [49]  [49]  [49]  [49]  This study  This study  This study  [50]  [51]  [52]  [53]  [53]  [53]  This study  This study  This study  [43]  This study  This study  [54]  [55]  This study  [56]  [44]  A. Rietsch (Case Western Reserve Uni.)  [44]  [44]  A. Rietsch |

*Transposon mutants obtained from the University of Washington mutant bank [49] were transferred into our wild-type genetic background (PAO1) using the generalized transducing phage ΦPA3 as a vehicle [57] and were PCR-verified prior to use.
