# Supplementary figures and images for: Type III secretion system expression in oxygen-limited Pseudomonas aeruginosa cultures is stimulated by isocitrate lyase activity
Source: Open Biol. 2013 Jan;3(1):120131. doi: 10.1098/rsob.120131 (PMC3603453; doi:10.1098/rsob.120131)

Figure S1

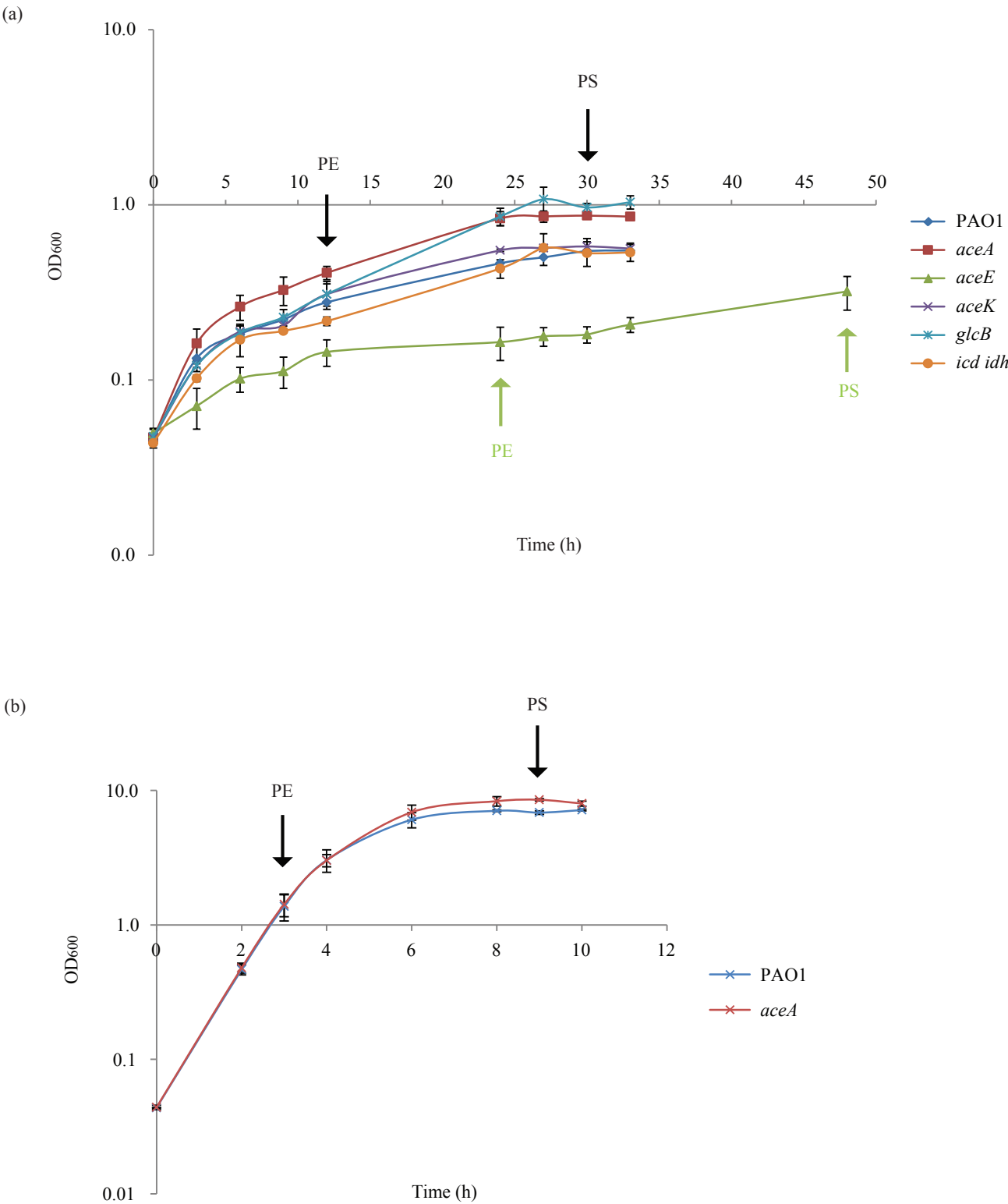

Supplement: Figure S1: Growth of TCA and glyoxylate cycle mutants. [file rsob120131-s2.pdf]

(a)

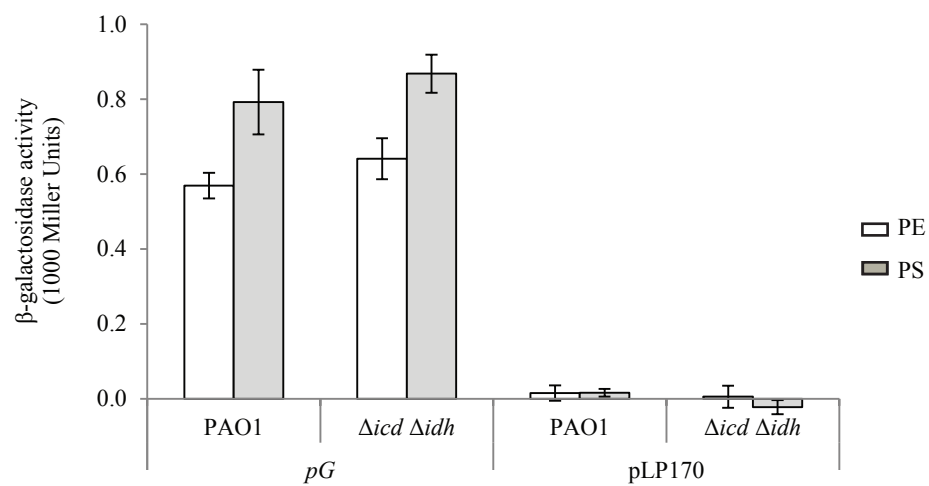

(b)

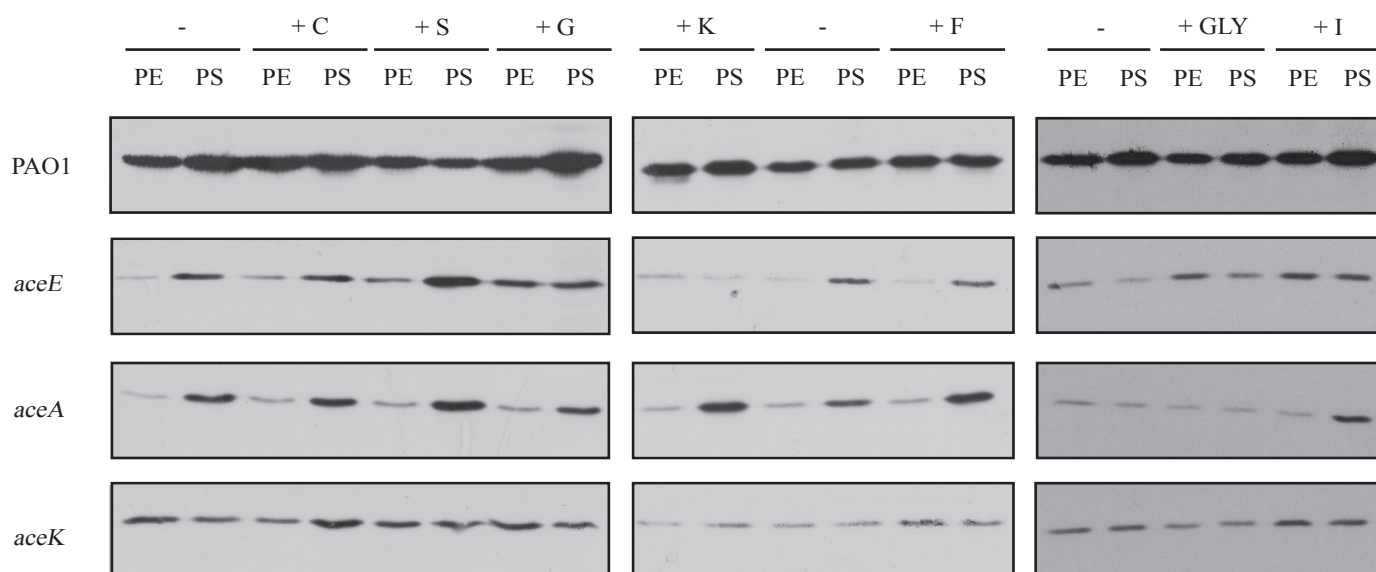

Supplement: Figure S2: T3SS expression and the TCA and glyoxylate cycles. [file rsob120131-s3.pdf]

Figure S3

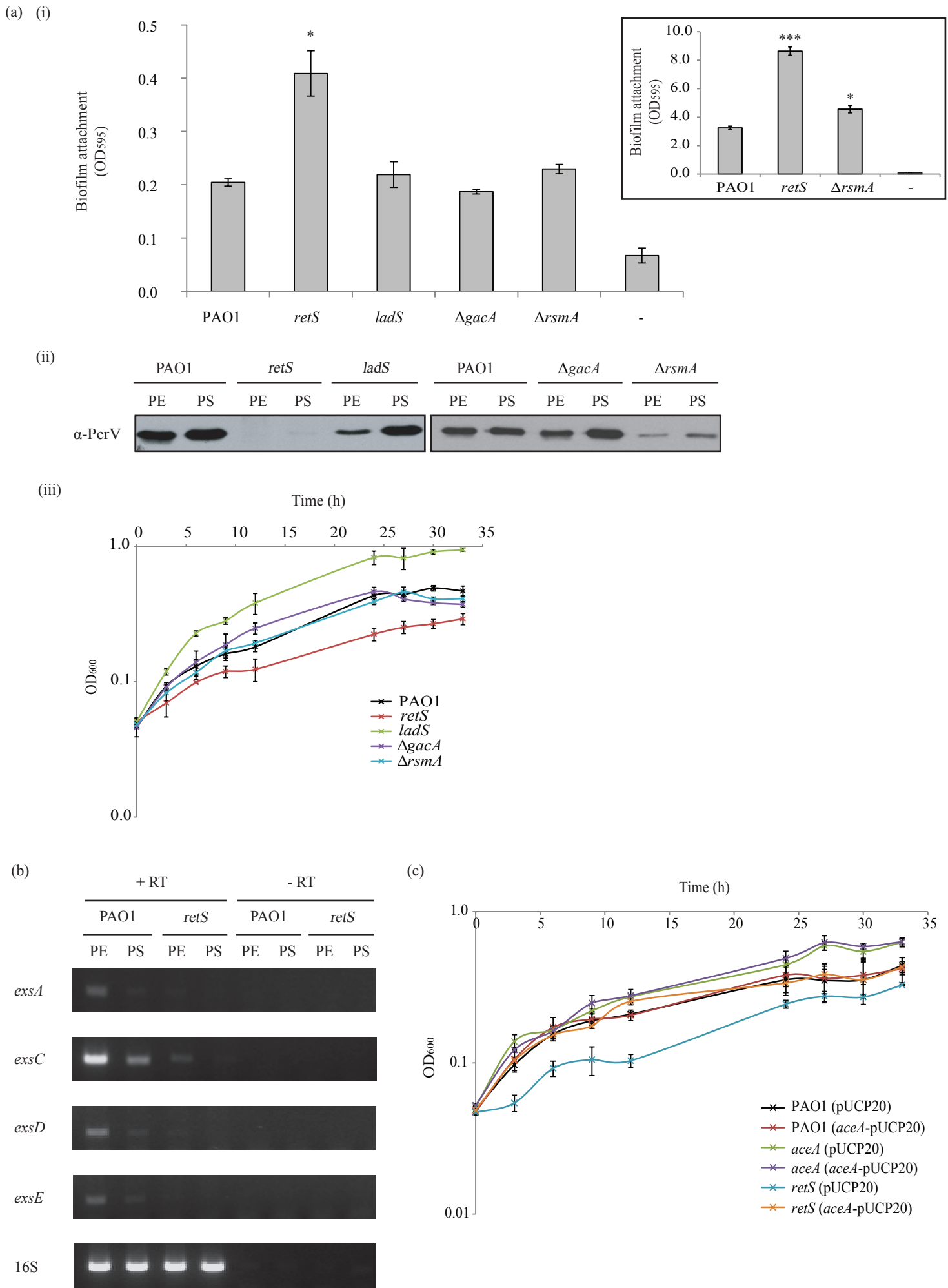

Supplement: Figure S3: T3SS expression and the RetS/LadS signalling pathways. [file rsob120131-s4.pdf]

Figure S4

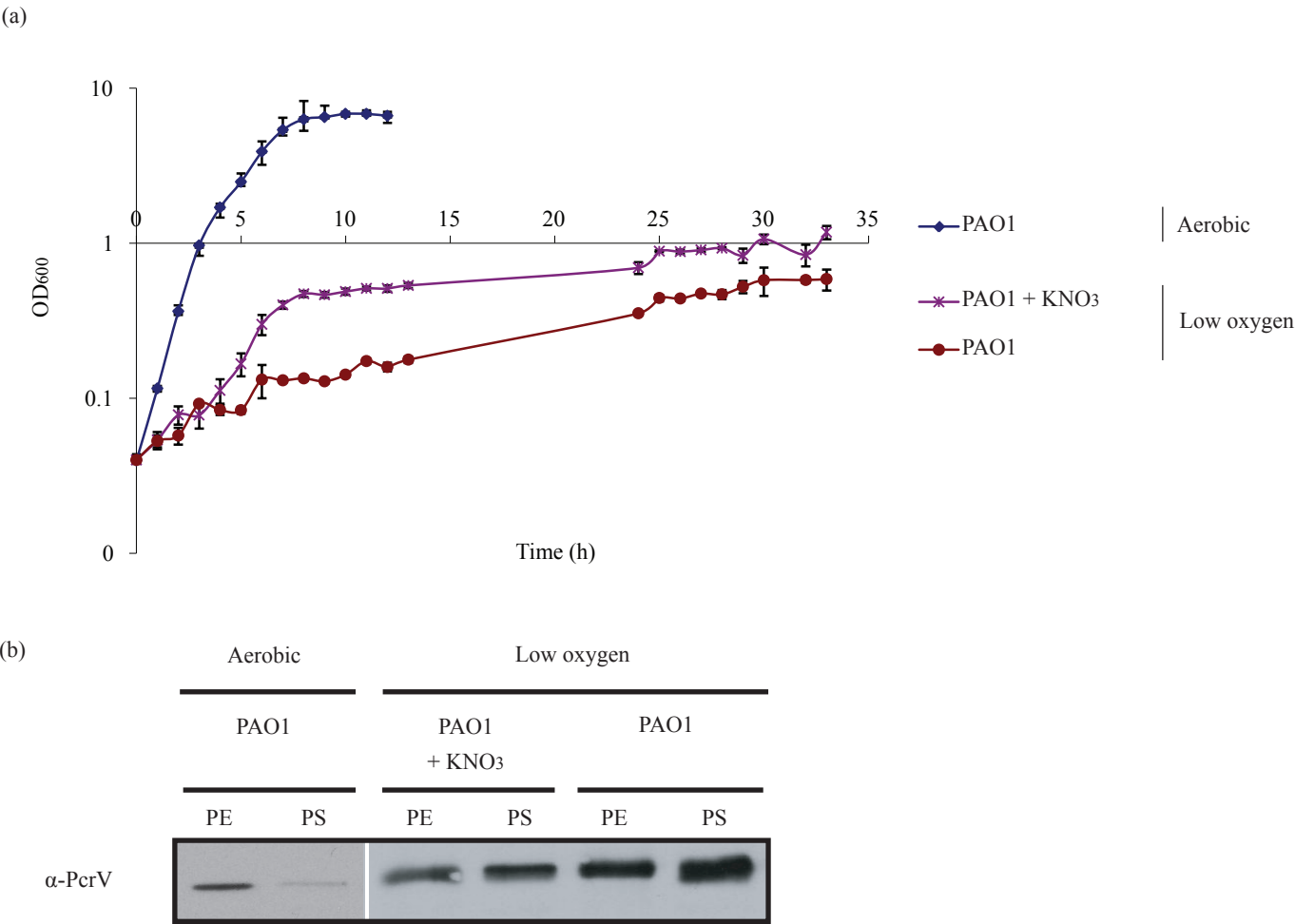

Supplement: Figure S4: Oxygen-limited cultures supplemented with KNO3. [file rsob120131-s5.pdf]
